# SCARB1 in extracellular vesicles promotes NPC metastasis by co-regulating M1 and M2 macrophage function

**DOI:** 10.1038/s41420-023-01621-9

**Published:** 2023-08-29

**Authors:** Wenhui Chen, Lili Bao, Qianqian Ren, Zixiang Zhang, Lu Yi, Wei Lei, Zhiyuan Yang, Yingna Lu, Bo You, Yiwen You, Miao Gu

**Affiliations:** 1 Department of Otorhinolaryngology Head and Neck Surgery, Affiliated Hospital of Nantong University, Nantong, Jiangsu Province China; 2 Institute of Otolaryngology Head and Neck Surgery, Affiliated Hospital of Nantong University, Nantong, Jiangsu Province China

**Keywords:** Cell death, Cancer

## Abstract

Distant metastasis is currently the main factor affecting the prognosis of nasopharyngeal carcinoma (NPC), and understanding the mechanisms of metastasis and identifying reliable therapeutic targets are critical for improving prognosis and achieving clinical translation. Macrophages, as important immune cells in the tumor microenvironment (TME), have been shown to regulate metastasis. And extracellular vesicles (EVs) secreted by stromal cells and tumor cells play the important role in intercellular communication in the tumor microenvironment. However, the role of NPC-EVs on macrophages and their function in regulating macrophages to affect metastasis has not been fully clarified. In this study, we report that NPC-EVs can be uptake by macrophages and alter macrophage polarization, for the first time, we identified the genes implicated in these regulatory functions: SCARB1, HAAO, and CYP1B1. Moreover, we found that SCARB1 was positively associated with metastasis and poor prognosis of NPC. Interestingly, we found that SCARB1-rich EVs promoted M1 macrophages ferroptosis to decrease M1 macrophages infiltration by upregulating the HAAO level while decreasing phagocytosis of M2 macrophages by upregulating the CYP1B1 level. Finally, we identified the SCARB1-binding gene KLF9, which is involved in the transcription of HAAO and CYP1B1. Our findings showed that SCARB1-EVs promoted metastasis by co-regulating M1 and M2 macrophage function. The related mechanism will provide a new therapeutic strategy to help patients with NPC improve their prognosis.

## Introduction

Nasopharyngeal carcinoma (NPC) is cancer that develops from the epithelial cells of the nasopharynx and is prevalent in southern China [1, 2]. Because of its hidden location, most patients with NPC are diagnosed at an advanced stage of the disease [3, 4]. The NCCN guidelines illustrate that the radiotherapy-chemotherapy combination is the treatment of choice for regionally advanced NPC and can significantly reduce local recurrence rates [5]. Nevertheless, distant metastasis remains the main cause of therapy failure and the cause of most NPC-related deaths [6]. As a result, it is crucial to investigate the molecular mechanisms that lead to the metastasis of NPC to formulate more effective treatment options.

Extracellular vesicles (EVs) are bilayer membrane vesicles that act as molecular cargo transporters and manage intercellular communication, leading to the metastasis of many malignancies [7, 8]. EVs have highly specific fusion properties and a mechanism of uptake into receptor cells that can focus on regulating the tumor microenvironment (TME), leading to metastasis [9, 10]. Tumor cells express unique integrins via secreted EVs that then target organ-specific cells, thereby enhancing metastatic colonization [10]. NPC-EVs were found to be closely related to metastasis in our previous study [11, 12]. However, the potential mechanisms of EV-induced metastasis are mostly uncharted and require further investigation.

Macrophages, a key cell type in tumor microenvironments, are critical for immunological homeostasis. Signals from the microenvironment activate and polarize them, resulting in classically activated (M1) and alternatively activated (M2) phenotypes [13]. However, the mechanisms underlying macrophage polarization remain largely unknown in NPC. Macrophages are the body’s primary iron storage cells, and their ferroptosis directly influences metastasis regulation, altering disease prognosis. Ferroptosis is an iron-dependent controlled necrosis subroutine characterized by enhanced lipid peroxidation due to a lack of activity of the glutathione peroxidase 4 enzyme (GPX4), which requires glutathione to function [14–16]. Through a variety of receptor-ligand interactions, macrophages, the body’s intrinsic immune cells, can remove host pathogens and dead cells. They patrol the host for unwanted targets and are removed via phagocytosis and digestion when found. Macrophages remove a wide variety of targets, including dead or dying host cells, antibody-conditioned cells, and specific pathogens [17].

Scavenger receptor class B type I, also known as SCARB1 or CLA-1 (CD-36 and LIMPII analog 1), is a membrane protein with a molecular weight of 82 kDa that acts as the major HDL receptor and mediates the selective uptake of HDL cholesterol by cells [18]. SCARB1 has been reported to be overexpressed as an oncogene in a range of cancers, including prostate cancer [19, 20], breast cancer [21–24], and adrenal cortical cancer [25]. We characterized the expression level of SCARB1 in NPC and its relationship with prognosis by tissue microarray, and subsequent experiments demonstrated that SCARB1 promoted metastasis through the regulation of HAAO in M1 macrophages and CYP1B1 in M2 macrophages by the transcription factor KLF9, respectively.

The main purpose of this article was to clarify the molecular mechanisms by which EVs of the NPC microenvironment promote metastasis through the combined regulation of ferroptosis in M1 macrophages and phagocytosis in M2 macrophages, this provided a novel idea and focus for the improvement of NPC prognosis.

## Results

### NPC EVs influence macrophage polarization and function

**Fig. 1:**
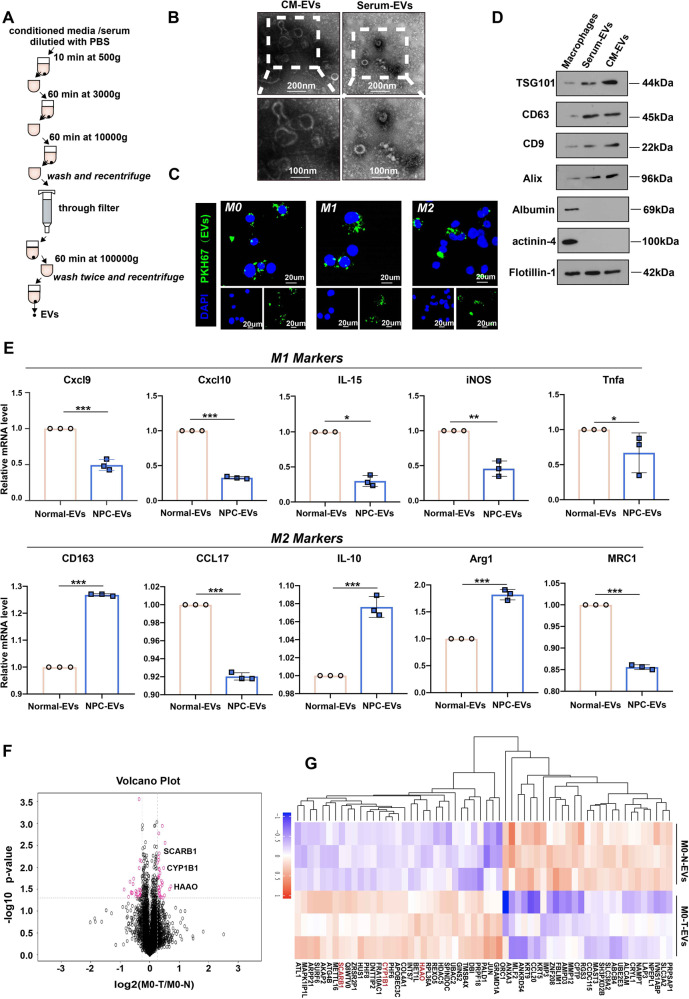
NPC- EVs influence macrophage polarization and function. **A** Flow chart of steps of EV extraction. **B** Representative electron microscopy images of CM-EVs and Serum EVs. Scale bar:100 nm. **C** Uptake of EVs in macrophages by confocal microscopy. Blue: DAPI staining; green: PKH-67-labeled EVs. Scale bar: 20 μm. **D** Western blotting analysis for CD63, CD9, Alix, Flotillin 1, TSG101, Albumin, and actinin-4 in macrophages and EVs. **E** Macrophage polarization was detected by qRT-PCR, Normal-EVs: EVs from normal; NPC-EVs: EVs from NPC patients. (Student’s *t*-test, *P < 0.05, **P < 0.01, ***P < 0.001). **F** Differential gene volcanoes of M0 macrophages after co-culture with EVs from normal (M0-N) and EVs from NPC patients (M0-T), respectively. **G** Gene clustering for differential macrophage expression. M0-N-EVs: M0 macrophages after co-culture with EVs from normal; M0-T-EVs: M0 macrophages after co-culture with EVs from NPC patients.

It has been reported that macrophages play an important role in the tumor microenvironment [26–28], but whether they have the same effect on primary site tumors and metastases is unknown. We inoculated the human NPC cell line CNE2 cells subcutaneously into the axillae of nude mice (Fig. S1A). Macrophage-depleting agent and its control were injected intraperitoneally. It was found that the presence of macrophages did not affect the volume of the tumor at the subcutaneous site (Fig. S1B, C). Meanwhile, the same grouping in the tail vein injection model in nude mice showed different results (Fig. S1D), the number of lung metastatic nodules was significantly reduced after macrophage depletion compared to the control group (Fig. S1E, F). This finding suggested that macrophages can promote tumor metastasis in vivo but do not affect tumor growth at the primary site. Under physiological conditions, macrophages are polarized towards the pro-inflammatory anti-tumor M1 type (classically activated). In contrast, macrophages are polarized towards the anti-inflammatory and tissue remodeling M2 type in the tumor microenvironment. Next, we co-cultured the macrophages with CNE2 cells in the Transwell chambers (pore size 0.4 μm) (Fig. S1G). CNE2 cells from the lower chamber were digested and used in subsequent experiments after 48 h of co-culture. The presence of M2 macrophages conferred a greater migratory and invasive capacity to CNE2 cells, whereas M1 macrophages played an opposed role (Fig. S1H–M). It is worthwhile to consider and investigate what factors in the tumor are responsible for such a result. EVs are bridges and transport carriers that connect multiple cells, making them important members of the tumor microenvironment. As a result, we speculate whether NPC-derived EVs endow macrophages with the ability to promote the malignant process. We then extracted EVs of conditioned medium of CNE2 cells, and serum from patients with NPC (Fig. 1A), characteristic lipid bilayers of EVs could be observed by transmission electron microscopy (Fig. 1B). Serum EVs from patients with NPC can be efficiently taken up by macrophages as observed by laser confocal microscope (Fig. 1C). These EVs were also characterized by marker expression (Fig. 1D). After co-culturing EVs from patients with NPC and M0 macrophages for 48 h, we measured the mRNA levels of macrophage markers and discovered that the expression levels of M1 markers decreased collectively (Fig. 1E). This means that NPC-derived EVs cause macrophages to polarize in a pro-cancer direction, thus making the fight against cancer less potent. To investigate which genes in EVs affect macrophages, we co-cultured EVs from three NPC patients and normal with macrophages and then performed LC-MS/MS to determine the differentially expressed genes (Fig. 1F, G).

### SCARB1 is highly expressed in NPC-EVs, which upregulates the HAAO level in M1 and CYP1B1 level in M2

**Fig. 2:**
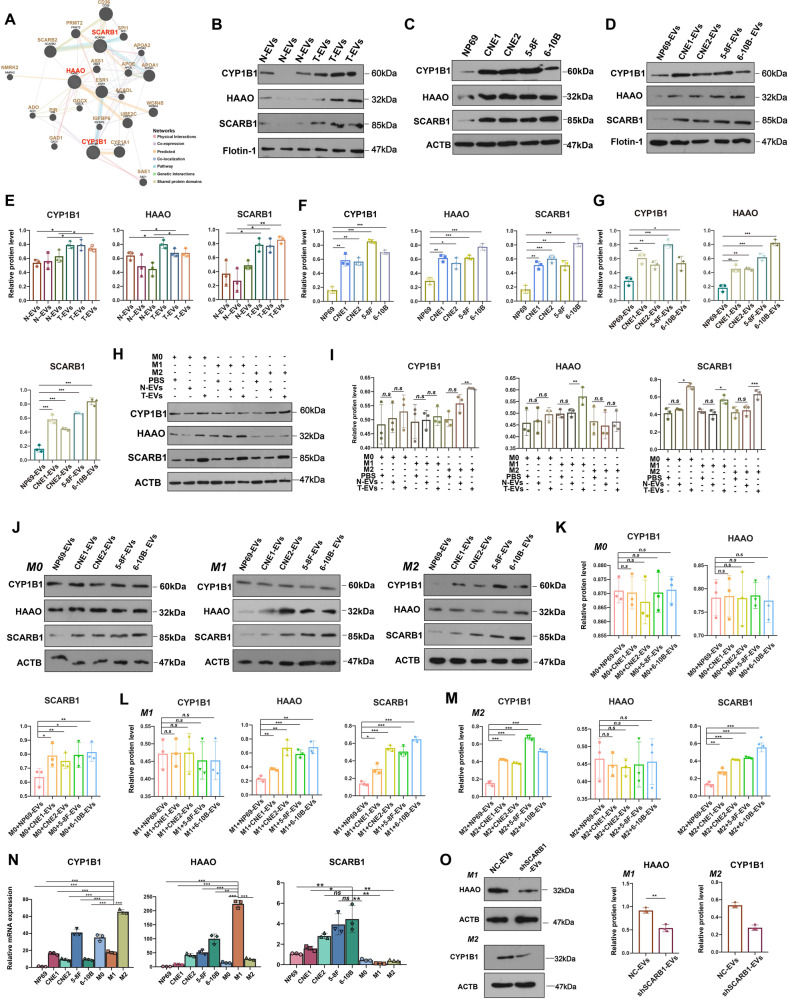
SCARB1 is highly expressed in NPC-EVs, which upregulates the HAAO level in M1 and CYP1B1 level in M2. **A** Interaction network of these three proteins in the Genemania database. **B**, **E** WB analysis of protein levels of CYP1B1, HAAO, and SCARB1 in serum-derived EVs from normal and patients with NPC and statistics. N-EVs: EVs from normal; T-EVs: EVs from NPC patients. **C**, **D**, **F**, **G** Analysis of CYP1B1, HAAO, and SCARB1 protein levels in NP69 and NPC cell lines and EVs. **H**, **I** Protein expression of CYP1B1, HAAO, and SCARB1 after co-culture of macrophages and EVs. **J**–**M** Analysis of CYP1B1, HAAO, and SCARB1 protein levels in M0, M1, and M2 macrophages and NP69 and NPC cell line-derived EVs, respectively. **N** Analysis of mRNA content of CYP1B1, HAAO, and SCARB1 in NP69, NPC cell lines, and M0, M1, and M2 macrophages. **O** Protein levels of HAAO after co-culture of SCARB1-silencing EVs in M1 macrophages and CYP1B1 in M2 macrophages were determined (Student’s *t*-test, *P < 0.05, **P < 0.01, ***P < 0.001).

Among the many genes whose expression was upregulated, three genes caught our attention, namely SCABR1, HAAO, and CYP1B1 (Fig. 1F). Firstly, it has been reported that these three genes are closely related to the function of macrophages [29–32], and secondly, we discovered via the Genemania database that SCARB1 could influence the expression of HAAO and CYP1B1, with an interaction between the latter two (Fig. 2A). Following that, Western Blotting experiments confirmed that the expression of SCARB1, HAAO, and CYP1B1 was all higher in EVs of NPC patients than in normal (Fig. 2B, E), which was consistent with the LC-MS/MS results. They were also significantly more highly expressed in NPC cell lines (Fig. 2C, F) and EVs (Fig. 2D, G) than in normal. Surprisingly, we discovered that SCARB1 expression was high in all M0, M1, and M2 cells after co-culture with NPC-EVs, whereas HAAO and CYP1B1 expression was only increased in M1 and M2 macrophages after co-culture with NPC-EVs, respectively (Fig. 2H, I). The same results were obtained when co-cultivating human normal nasopharyngeal epithelial cell line NP69 and NPC cell lines released EVs with M0, M1, and M2 macrophages, respectively (Fig. 2J–M). We then considered whether SCARB1 in EVs originating from NPC affected the expression of HAAO and CYP1B1 in macrophages. To back up our suspicions, we used qRT-PCR to test the mRNA expression of the three and discovered that the level of SCARB1 was significantly higher in NPC cell lines than in macrophages (Fig. 2N), whereas HAAO was highly expressed in M1 macrophages and CYP1B1 in M2 macrophages (Fig. 2N). We also co-cultured M1 and M2 macrophages with SCARB1-knockdown EVs released by CNE2 cells and discovered that SCARB1 knockdown reversed HAAO and CYP1B1 overexpression in M1 and M2 macrophages, respectively (Fig. 2O). Such a result confirmed our suspicion that SCARB1 in EVs of NPC was found to be transmitted to macrophages, causing an increase in HAAO in M1 macrophages and CYP1B1 in M2 macrophages. According to the data presented above, the role of EVs in regulating macrophage polarization to influence NPC metastasis is most likely due to EVs-carried SCARB1 regulating HAAO/CYP1B1 in macrophages.

### The expression level and clinical significance of SCARB1 in NPC

**Fig. 3:**
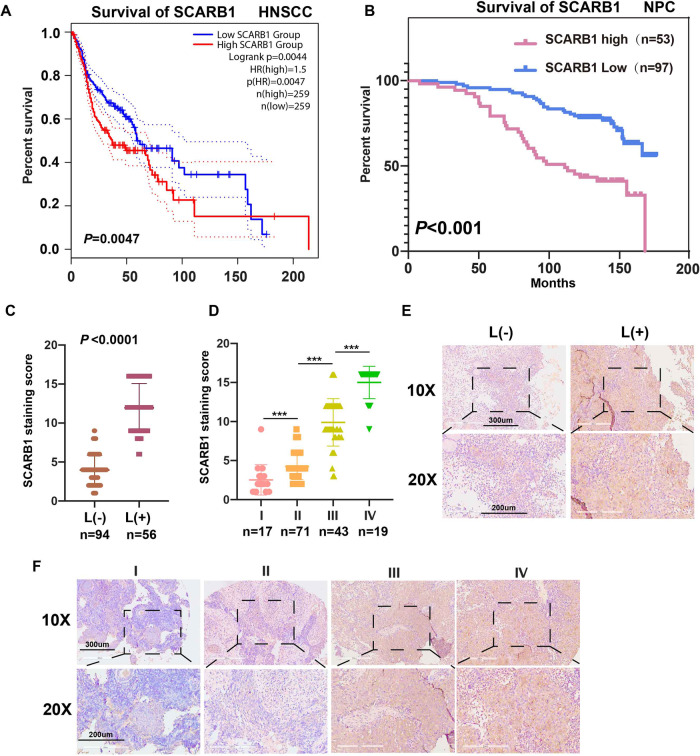
The expression level and clinical significance of SCARB1 in NPC. **A** SCARB1 survival in HNSCC in GEPIA2. **B**–**D** Statistical analyses of the association of SCARB1 expression with prognosis, lymph node metastasis, and tumor stage. ***P < 0.001. **E**, **F** Representative tissue microarrays images of SCARB1 staining in tissues.

Since SCARB1 was found to be significantly overexpressed in NPC cell lines, we speculate that high expression of SCARB1 may be closely associated with the malignant progression of NPC. Based on the GEPIA2 database, we found that patients with high SCARB1 expression had a significantly poorer prognosis in the human head and neck squamous carcinoma (HNSCC) (Fig. 3A). In addition, the higher the expression level of SCARB1, the worse the prognosis of NPC patients based on the tissue microarrays (Fig. 3B). In Table S1, the relevant clinical data of the tissue microarrays were categorized and summarized. In brief, elevated SCARB1 expression was significantly associated with lymph node metastasis (Fig. 3C, E), tumor stage (Fig. 3D, F), and the survival rate of patients (Fig. 3B).

### SCARB1-EVs promote M1 macrophages ferroptosis by increasing HAAO level

**Fig. 4:**
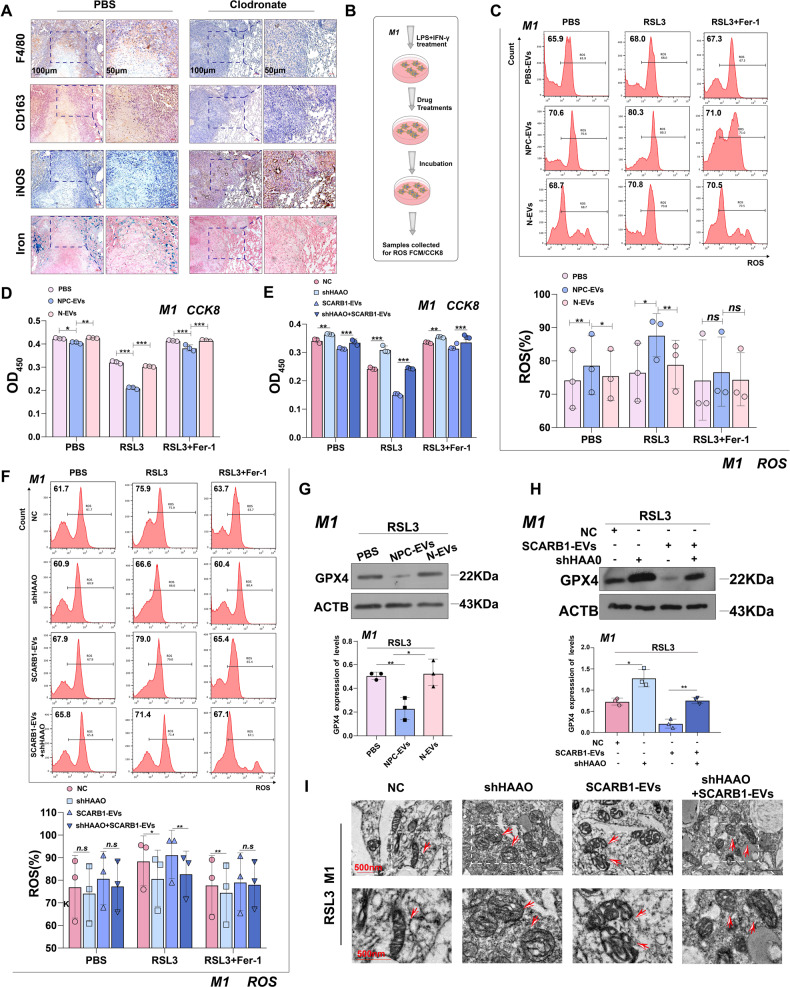
SCARB1-EVs promote M1 macrophages ferroptosis by increasing HAAO level. **A** IHC of lung metastases in the depleting agent group and the control group. **B** Flow chart of the mechanism of CCK8 and flow ROS assay on M1 macrophages. **C**, **F** Flow cytometry of ROS in macrophages (Two-way ANOVA). **D**, **E** Cell death was estimated by CCK8 assay (two-way ANOVA). EVs were derived from CNE2 cells. All graphs show the mean ± SEM of at least three independent experiments. *P < 0.05, **P < 0.01, ***P < 0.001. **G**, **H** WB analysis of the GPX4 expression levels. **I** Representative transmission electron microscopy (TEM) images of macrophages.

To investigate the potential effects of EVs carried SCARB1 on macrophage function via HAAO/CYP1B1 regulation. We first investigated the role of SCARB1-mediated HAAO on M1 macrophage function. The Immunohistochemistry (IHC) of lung tissues from the macrophage depletion group and the control group was compared (Fig. S1D, Fig. 4A). The macrophage-depleting regent could effectively reduce the number of macrophages in mice, as shown by the stronger expression of F4/80 in the control group compared to the depleted group (Fig. 4A). Conversely, the expression of CD163, a marker of M2 macrophages, was higher in the control group compared to the depleted group (Fig. 4A). The control group also showed reduced expression of iNOS, an M1-type marker (Fig. 4A), compared to CD163. It is also interesting that after depletion, the level of iron infiltration by macrophages, the body’s primary iron storage cells, decreased (Fig. 4A). Since HAAO has been reported to be involved in iron metabolism in studies and is increased by the existence of SCARB1-rich EVs in M1 macrophages. Following that, we attempted to test macrophage sensitivity to a pro-ferroptosis treatment with a specific GPX4 inhibitor, RSL3 (1S,3R)-2-(2-chloroacetyl)-2,3,4,9-tetra-hydro phenyl-1-(methoxycarbonyl) Indole-3-carboxylic acid (1H-pyrido[3,4-b] indole-3-carboxylic acid). Serum EVs from NPC patients enhanced ROS levels in M1 macrophages in the presence of RSL3 (Fig. 4B, C). However, ferrostatin-1 (Fer-1), a selective ferroptosis inhibitor, could effectively prevent the increase in ROS caused by RSL3 (Fig. 4C). EVs in the serum of NPC patients rendered M1 macrophages vulnerable and lost viability, according to the results of CCK8 experiments (Fig. 4D). Furthermore, knocking down of HAAO reduced the ROS levels of M1 macrophages, and SCARB1-rich EVs secreted by CNE2 cells could rescue this result (Fig. 4F), confirming that SCARB1 in NPC-EVs increased ferroptosis of M1 macrophages via HAAO. CCK8 Assay and GPX4 expression both supported the same conclusion (Fig. 4E, G, H). Also, according to mitochondrial electron micrographs, SCARB1 increased cellular mitochondrial membrane density, a hallmark of ferroptosis (Fig. 4I). All these findings indicate that SCARB1 in NPC-EVs promotes ferroptosis in M1 macrophages, thereby weakening the protective force against tumors and promoting NPC progression.

### SCARB1-EVs inhibit M2 macrophage phagocytosis by upregulating the CYP1B1 level

**Fig. 5:**
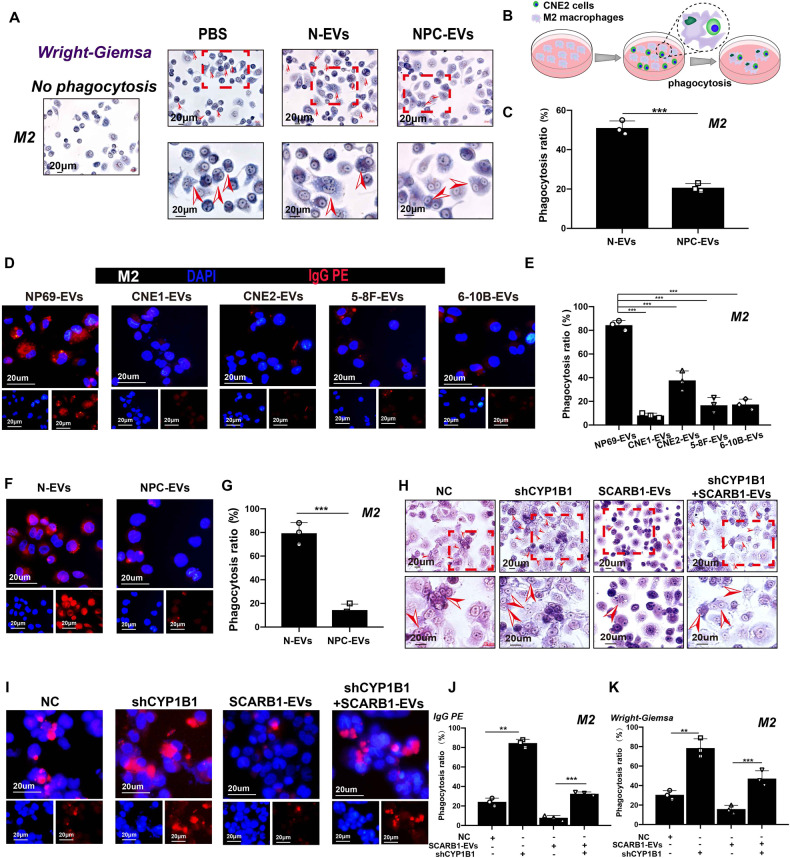
SCARB1-EVs inhibit M2 macrophage phagocytosis by upregulating the CYP1B1 level. **A** Wright-Giemsa image of macrophages after phagocytosis of CNE2. N-EVs: EVs from the serum of normal; NPC-EVs: EVs from the serum of NPC patients. The arrow represents M2 macrophages after phagocytosis of CNE2 cells. **B** Diagram of macrophage phagocytosis of CNE2. **C** Statistics of devouring ability. **D**, **E** Phagocytosis map and statistics of latex beads after co-cultivation of macrophages with EVs of NP69 and NPC cell lines origin. **F** Images of macrophages engulfing latex beads. **G** Statistics of devouring ability. **H** Staining diagram of M2 macrophages with Wright-Giemsa. The arrow represents M2 macrophages after phagocytosis of CNE2 cells. **I** Images of macrophages engulfing latex beads. EVs were derived from CNE2 cells. **J**, **K** Statistics of devouring ability (All graphs show the mean ± SEM of at least three independent experiments. **P < 0.01, ***P < 0.001).

Then, we studied how SCARB1 in EVs affected M2 macrophage function by regulating CYP1B1. Since the function of CYP1B1 has been reported to be related to phagocytosis and specifically elevated significantly in M2 macrophages, we investigated the effect of NPC-EVs on M2 macrophage phagocytosis. The Wright-Giemsa staining results revealed that M2 macrophage phagocytosis was significantly reduced after co-cultivation with NPC-EVs. The arrow represents M2 macrophages after phagocytosis of CNE2 cells (Fig. 5A–C). Meanwhile, M2 macrophages co-cultured with EVs from NPC cell lines show impaired phagocytosis of latex beads (Fig. 5D, E), and EVs derived from patient serum did the same (Fig. 5F, G). Notably, SCARB1 can be delivered to M2 macrophages and increase CYP1B1 expression, whereas SCARB1-rich EVs decreased phagocytosis in M2 macrophages, and rescue experiments confirmed that SCARB1 inhibited the enhanced phagocytosis caused by CYP1B1 knockdown (Fig. 5H–K). M2 macrophages play an important role in the tumor microenvironment as tumor-promoting “accomplices” and when their phagocytic capacity is compromised, the cancer-promoting effect is amplified.

### SCARB1-EVs promote metastasis by co-regulating HAAO in M1 and CYP1B1 in M2

**Fig. 6:**
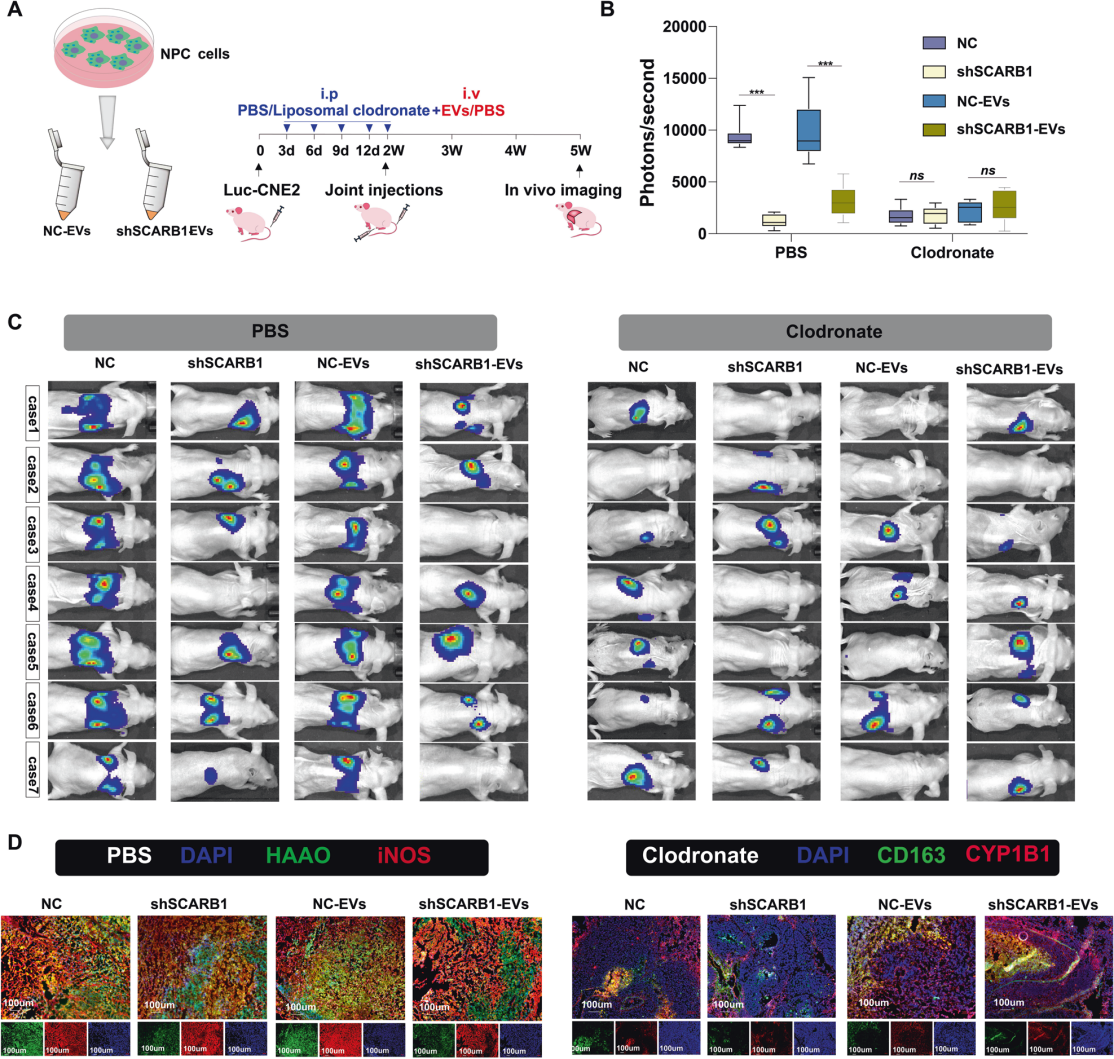
SCARB1-EVs promote metastasis by co-regulating HAAO in M1 and CYP1B1 in M2. **A** Schematic diagram of tail vein injection protocol of EVs and CNE2 cells. **B**, **C** Visualization of lung metastasis in Balb/c nude mice (***p < 0.001, one-way ANOVA). **D** Immunofluorescence of lung section tissue.

To examine the effect of SCARB1-rich EVs in metastasis in vivo. We injected CNE2 cells knocked down with SCARB1 lentivirus and EVs through the tail vein, and discovered that lung metastasis was significantly reduced in mice. However, when macrophages were depleted, the inhibitory effect of SCARB1 inhibition on metastasis was lost, demonstrating that SCARB1 promotes NPC metastasis via the presence of macrophages (Fig. 6A–C). Furthermore, immunofluorescence results confirmed the co-localization of HAAO with the M1 marker iNOS and CYP1B1 with the M2 marker CD163 (Fig. 6D), confirming that SCARB1 was regulated in vivo by combining HAAO in M1 and CYP1B1 in M2. When two major anti-cancer strategies are combined, they can significantly delay NPC metastasis in the body.

### SCARB1 regulates HAAO and CYP1B1 levels via the transcription factor KLF9

**Fig. 7:**
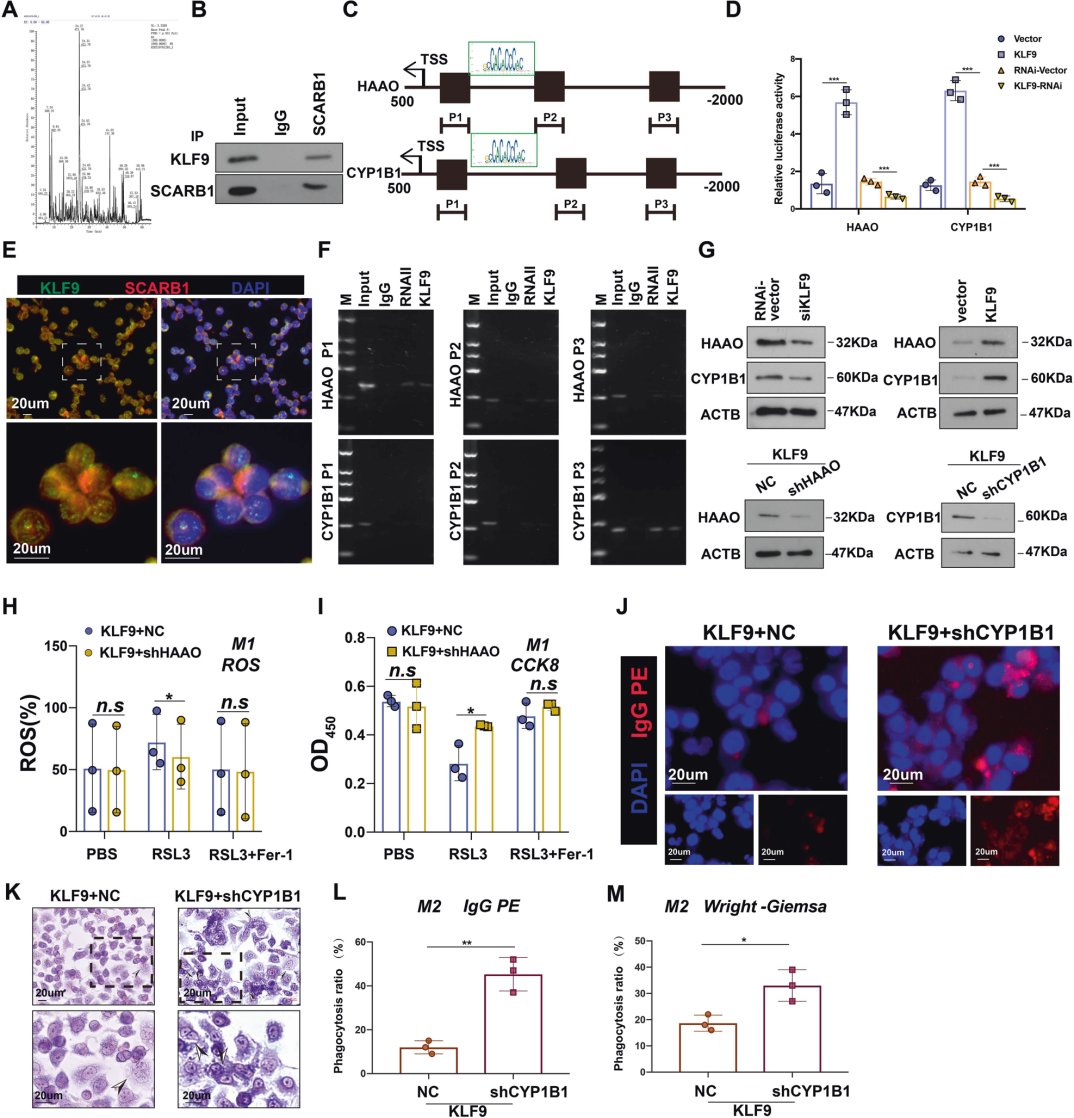
SCARB1 regulates HAAO and CYP1B1 levels via the transcription factor KLF9. **A** Base peak plot of the IP mass spectra of SCARB1. **B** IP experiments to validate the binding of SCARB1 to KLF9. **C** Prediction of possible KLF9-target genes. **D** Luciferase reporter assay of HAAO and CYP1B1 transcriptional activity. **E** Cellular immunofluorescence co-localization of SCARB1 with KLF9. **F** ChIP assay for validation of possible KLF9-target genes. **G** HAAO and CYP1B1 protein levels in the cells. **H** ROS flow statistics of M1 macrophages (two-way ANOVA). **I** Statistics of CCK8 assay in M1 macrophages (Two-way ANOVA). **J** Images of M2 macrophages engulfing latex beads. **K** Staining diagram of M2 macrophages with Wright-Giemsa. The arrow represents M2 macrophages after phagocytosis of CNE2 cells. **L**, **M** Phagocytosis statistics of M2 macrophages (*p < 0.05, **P < 0.01, ***p < 0.001, Student’s *t*-test).

The IP mass spectrometry of macrophages confirmed that SCARB1 could bind the transcription factor KLF9 (Fig. 7A, B), and the transcription factor database JASPER revealed that KLF9 had binding sites to the downstream HAAO and CYP1B1 (Fig. 7C). KLF9 overexpression increased HAAO/CYP1B1 promoter-driven reporter activity significantly, whereas silencing KLF9 had the opposite effect (Fig. 7D), confirming that KLF9 directly targets and positively regulates HAAO and CYP1B1. Immunofluorescence studies also showed that the transcription factor KLF9 co-localized with SCARB1 in the cytoplasm (Fig. 7E). The chromatin immunoprecipitation (ChIP) assay confirmed that KLF9 can directly bind to the promoters of HAAO and CYP1B1 (Fig. 7F). Furthermore, silencing KLF9 significantly reduced the protein levels of HAAO and CYP1B1, whereas overexpression of KLF9 had the opposite result (Fig. 7G). Then, we studied whether HAAO or CYP1B1 activation was required for the ability of KLF9 to regulate macrophage function, silencing HAAO reduced the level of ROS in KLF9-overexpressing M1 macrophages, preventing ferroptosis of M1 macrophages (Fig. 7H), meanwhile, silencing of HAAO enhanced the viability of KLF9-overexpressing M1 macrophages (Fig. 7I); silencing CYP1B1 increased the level of phagocytosis in KLF9-overexpressing M2 macrophages (Fig. 7J–M). Taken together, these findings suggested that activation of HAAO or CYP1B1 was required for KLF9 to regulate macrophage function and that KLF9 increased the tumorigenicity of NPC by directly targeting and positively regulating HAAO and CYP1B1.

### SCARB1-EVs regulate HAAO and CYP1B1 to promote NPC metastasis via KLF9

**Fig. 8:**
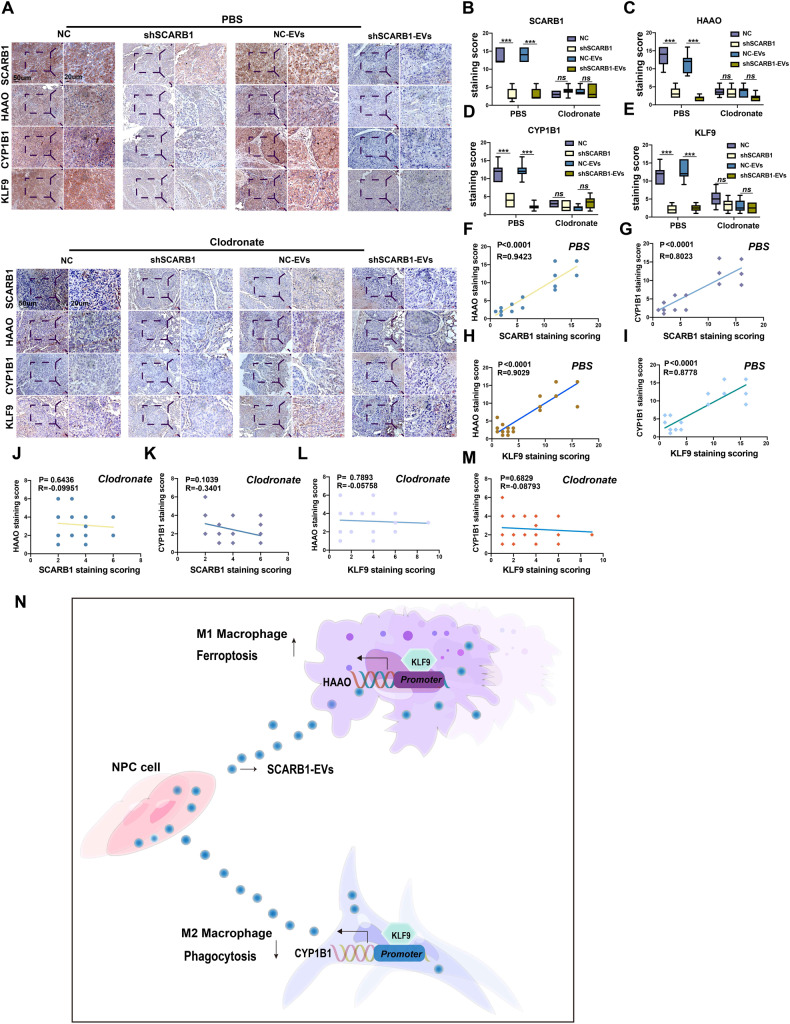
SCARB1-EVs regulate HAAO and CYP1B1 to promote NPC metastasis via KLF9. **A** HAAO and CYP1B1 expression levels were associated with SCARB1and KLF9 expression in the presence of macrophages. **B**–**E** expression using one-way ANOVA (***P < 0.001). **F**, **G**, **J**, **K** Pearson correlation between HAAO, CYP1B1, and SCARB1 expression. **H**, **I**, **L**, **M** Pearson correlation between HAAO, CYP1B1, and KLF9 expression. **N** Mechanisms by which SCARB1-rich EVs promote metastasis through regulation of M1 macrophage ferroptosis and M2 macrophage phagocytosis by transcription factor KLF9, respectively.

IHC results showed that the expression of HAAO and CYP1B1 was also reduced injected with SCARB1-silencing cells and EVs, the same held for the interactions between KLF9, HAAO, and CYP1B1, but this correlation was lost after macrophage depletion (Fig. 8A–M). In summary, we discovered that EVs secreted by NPC could alter macrophage function and promote metastasis; EVs secreted by NPC with high SCARB1 expression increased HAAO expression in M1 macrophages, leading to increased ferroptosis of M1 macrophages and decreased infiltration in the tumor microenvironment; meanwhile, SCARB1 in EVs increased CYP1B1 expression in M2 macrophages, reducing phagocytosis, and both promoted metastasis synergistically (Table 1).

**Table 1:** Relationship between SCARB1 expression and clinical-pathological factors of NPC.

Clinical indicators	Total	Low scoring	High scoring	*R*^2^	*P*
T stage					
T1–T2	137	88	49	0.2728	<0.0001
T3	17	9	8		
N stage					
N0–N1	141	97	44	0.1422	<0.0001
N2–N3	9	1	8		
Grade					
I–II	88	86	2	0.3396	<0.0001
III–IV	62	12	50		
Prognosis					
Dead	53	23	30	0.1487	<0.0001
Alive	97	75	22		

## Discussion

The current focus of macrophage research is on how to use the properties of M1 and M2 macrophages against tumors. Our study found that high expression of SCARB1 in EVs of NPC increased HAAO expression in M1 macrophages, resulting in increased ferroptosis and decreased infiltration of M1 macrophages; meanwhile, SCARB1 in EVs increased CYP1B1 expression in M2 macrophages to decrease phagocytosis of NPC cells by M2 macrophages. Ultimately, the combination of the two together affected macrophage function and promoted NPC metastasis. The related mechanism will provide a new therapeutic strategy to improve the prognosis of patients with NPC.

The Krüppel-like factor family of TFs regulates diverse biologic processes, including cell proliferation, differentiation, migration, and apoptosis [33, 34]. Krüppel-like factor 9 (KLF9) was previously known as a basic transcription element binding protein due to its specific binding to the basic transcription element, a GC box of CYP1A1 gene promoter region [35]. In the past few years, KLF9 has been reported to be associated with colorectal, breast, prostate, and liver cancers [36–39]. However, in contrast to other tumors, KLF9 plays a role in NPC that promotes tumor progression. HAAO (3-Hydroxyanthranilate 3,4-dioxygenase) is a monomeric cytosolic protein belonging to the family of intramolecular dioxygenases containing nonheme ferrous iron. It is widely distributed in peripheral organs, such as the liver and kidney, and is also present in low amounts in the central nervous system. HAAO has been reported to be associated with endometrial cancer and prostate cancer, but it has not been reported in NPC. CYP1B1 (cytochrome P450, family 1, subfamily B, polypeptide 1) is an enzyme that catalyzes the conversion of 17β-estradiol into 4-OHE2 [40]. CYP1B1 is abnormally highly expressed in various cancers in various cancers (e.g., breast, head and neck cancers and prostate cancer) [41–43]. Ju-Hong Jiang et al. found that CYP1B1 was abundantly enriched in human NPC tissues, but no report has yet elaborated on the detailed mechanism by which CYP1B1 promotes NPC progression. Our study found that SCARB1 in NPC-derived EVs could regulate CYP1B1 through the transcription factor KLF9 and inhibit phagocytosis of M2 macrophages, thus promoting NPC metastasis, and we will further explore the signaling pathways that play a role in this process in the future.

Cancer immunotherapy has been developed due to the gradual recognition that macrophages play an important role in tumor growth or regression. In human malignancies, M2-type macrophages predominate and release factors that promote tumor growth and metastasis but when M1-type macrophage expression is elevated, tumor progression can be significantly inhibited [44–46]. However, the reversal of M2 macrophages to M1 macrophages might reduce or stop cancer progression. The impacts include direct activation of M1 killer-type activity and the ability to stimulate Th1-type cytotoxic T cells and other effector cells via M1 macrophages. The discovery that macrophages can be modulated to play an important role in the fight against cancer is an exciting advance that appears to be on the verge of making successful cancer immunotherapy a reality. There are still challenges to be resolved. For example, early attempts to stimulate macrophages are accompanied by side effects [47], this has also been noted in recent attempts at immunotherapy [48]. Although the treatment of cancer is facilitated by increasing the M1 macrophage killing response, there is now a growing awareness that excessive killing and cytotoxic responses can lead to atherosclerosis and other chronic inflammatory diseases [49–51]. Therefore, in order to achieve optimal results in cancer and other conditions, it will be crucial to be aware of the powerful double-edged nature of the macrophage response.

Ferroptosis, an emerging mode of regulated cell death, is closely related to iron metabolism, lipid metabolism, amino acid metabolism, and other metabolic pathways. After intensive research on ferroptosis, scientists have made significant progress in both the molecular mechanisms of ferroptosis and disease treatment. However, the specific mechanism of ferroptosis leading to cell death is still unclear. Currently, the extent of ferroptosis is mainly reflected by detecting the level of peroxisomal phospholipids, which is mainly due to the fact that excessive peroxisomal phospholipids can cause membrane damage and even pore formation, compromising membrane integrity and ultimately leading to cell death [15]. A study found that lipid cross-linking may be an important factor in ferroptosis, the main reason may be that crosslinked lipids reduce the mobility of membrane components, leading to the failure of important functions associated with membranes and causing cell death [52]. In addition, it has been suggested that peroxidized PUFA is also capable of causing cell death by disrupting macromolecules that are closely related to cellular activity and thus causing cell death [53]. Therefore, the specific molecular mechanisms that mediate ferroptosis are still unclear and are an important direction for future research.

Professional phagocytes’ life and biology (macrophages and microglial cells are linked to dangerous pro-oxidant environments) [54], despite this, they are conspicuously absent from lists of cells that are susceptible to ferroptotic cell death [55, 56]. Given that their genomes contain sequences encoding for known ferroptotic machinery components [57, 58], we hypothesized that additional mechanisms may be responsible for phagocytes’ inability to carry out the ferroptotic program. Ferroptosis plays a dual role in tumorigenesis, both promoting and suppressing tumors, depending on the release of damage-associated molecular patterns triggered by ferroptosis injury and the activation of immune responses within the tumor microenvironment. Over the past period of time, researchers have become interested in studying the role of ferroptosis in cancer and applying this knowledge to improve cancer prevention, diagnosis, prognosis, and treatment. Ferroptosis play a complex role in the study of tumor biology and therapy. Developing translational anti-cancer strategies is a daunting task that requires ongoing research to explore the regulatory mechanisms of ferroptosis in greater depth. Exploring markers that help humans decipher ferroptosis will greatly improve patient prognosis, and this promises to be one of the hot spots for future research.

Two important innate immune functions of macrophages include phagocytosis of apoptotic cells, foreign bodies, and cancer cells, and processing of the phagocytosed material to present antigens to stimulate adaptive immunity [59]. Tumor-targeting antibodies, a potent class of anti-cancer biologics, act by directly inhibiting survival signaling mediated by NK cells and by inducing complement-dependent cytotoxicity (CDC) through complement cascade activation and promoting antibody-dependent cytophagy (ADCP) by macrophages [60]. Clinically approved anti-cancer monoclonal antibodies like rituximab and trastuzumab have been shown to exert their therapeutic effects primarily or partially via macrophage mediated ADCP [61, 62]. Malignant progression of tumors requires effective immune surveillance evasion as an adjunct [63]. Cancer cells are prevented from being phagocytosed by upregulating antibodies to CD47 on their surface, which interacts with signal-regulated protein alpha (SIRP) on macrophages to deliver the “don’t eat me” signal. In several cancer models, inhibition of the CD47-SIRP signaling axis with anti-CD47 antibodies or engineered SIRP-Fc fusions have been shown to restore the ability of macrophages to phagocytose cancer cells and to stimulate cytotoxic CD8+ T cell responses, thereby inhibiting the malignant progression of tumors [63, 64]. Thanks to its promising preclinical results, many clinical trials have been initiated in recent years to study various therapeutic variants such as anti-CD47 antibodies (Hu5F9-G4 and CC-90002), engineered high-affinity SIRP (ALX148) and SIRP-Fc fusion (TTI-621) [65, 66] We found that SCARB1 in NPC-derived EVs can inhibit macrophage phagocytosis and promote NPC metastasis by upregulating CYP1B1 in M2 macrophages, so targeting SCARB1 is likely to be a new target for NPC metastasis treatment.

Our findings have greatly contributed to our understanding of the role and mechanism of SCARB1 in regulating NPC. Thus, our findings provide a new therapeutic idea for the treatment of patients with NPC, which can be expected to greatly improve patient prognosis.

## Materials and methods

### Human NPC specimens and cell culture

The human NPC cell lines CNE-1 (highly differentiated), CNE2 (poorly differentiated), 5–8F (high tumorigenesis and metastasis), 6–10B (low tumorigenesis and metastasis), and the normal nasopharyngeal epithelial cell line NP69 were generously provided by Sun Yat-Sen University and Xiang-Ya School of Medicine. RPMI 1640 (Biological Industries Israel Beit-Haemek, 01-100-1ACS) containing 10% FBS (Biological Industries Israel Beit-Haemek, 04-001-1ACS) was used to culture NPC cells, while keratinocyte-SFM (Thermo Fisher Scientific, 17005-042) was used for NP69 cells.

All patients had not received any cancer therapies before the biopsy and were informed. Tufei Biotechnology examined SCARB1 expression in tissue microarrays. Table S1 contains a detailed list of patients’ clinicopathological features. SCARB1’s prognostic significance was determined using Kaplan-Meier analysis.

### Macrophage differentiation

THP-1 cells were treated for 1 day with 100 nM phorbol-12-myristate-13-acetate (PMA) before being differentiated into M1 macrophages with LPS (50 ng/mL, Sigma) + rIFN-γ (20 ng/mL, PeproTech, Rocky Hill, USA). IL-4 (20 ng/mL, PeproTech) was used to induce M2-polarized macrophages for 24 h.

### Transfection and transduction with lentiviral vectors

The lentiviral vectors and their corresponding controls were obtained from Shanghai Genechem Co., Ltd. qRT-PCR and western blotting were used to assess transfection efficiency. The particular vector used to clone shRNA control and shRNA was shown in Supplementary Data 2.

### Ferroptosis assay

Macrophages were co-cultured for 16 h with RSL3 (500 nM-1 M, MCE, HY-100218A) and Fer-1 (400–800 nM, MCE, HY-100579). The CCK8 assay was used to determine cell death. First, we inoculated the 96-well plates with cell suspension (100 μL/well) and placed the plates in an incubator for 24 h. Then, 10 μL of CCK8 solution was added to each well (taking care not to generate air bubbles); then the plates were incubated in the incubator for 2 h. Finally, the absorbance at 450 nm was measured using an enzyme marker. A dihydroethidium probe kit was used to measure intracellular ROS production in macrophages (BestBio). First, we aspirated the cell supernatant medium followed by centrifugation and then washed the cells once with PBS. Cells were collected and resuspended in diluted 37 °C pre-warmed working solution containing the probe at a cell concentration of 1 × 10^6^/ml. Cells are incubated for 45 min at 37 °C in a cell incubator protected from light. Mixing upside down every 3–5 min to bring the probe and cells into full contact. Then centrifuge, aspirate the stain, resuspend the cells with PBS (pH 7.4), and wash 3 times. This was done to fully remove the probe that had not entered the cells. Then the cells were resuspended with HBSS. Finally, flow cytometric detection was performed. The maximum excitation/emission wavelength is 510 nm/610 nm.

### Phagocytosis of latex beads assay

Macrophages were pre-inoculated on cell culture plates, after the medium was removed, the latex beads (Cayman, No.600540) diluted with RPMI 1640 complete culture at a dilution ratio of 1/500 were added. The cells were then placed in a cell incubator at 37 °C for 2 h, protected from light, washed 3 times with assay buffer, fixed with 4% paraformaldehyde, washed 3 times with PBS, and stained with DAPI (Beyotime, P0131), photographed by fluorescent microscopy.

### Phagocytosis of wright-giemsa assay

Macrophages were seeded in the 6-well plate first, after the macrophage medium was removed, 1.0 × 10^5^ CNE2/5–8F cells were resuspended well with 2 ml RPMI 1640 complete culture, after 2 h, removed and washed with PBS to remove unphagocytosed CNE2/5-8F cells, followed by adding 1 ml of Wright-Giemsa (Solarbio Science & Technology, G1020) solution to each well for 5 min.

### qRT-PCR evaluation

We added TRIzol (Thermo Fisher Scientific, 15596018) to the cells, then gently scraped the cells and collected them, after adding chloroform, followed by vortex and centrifugation to collect the uppermost layer, then added isopropanol, centrifuged, and collected the precipitate, and finally performed reverse transcription. After obtaining cDNA, qRT-PCR was performed.

### Animal model BALB/c nude mice

All mice were randomly grouped according to body weight. 1.0 × 10^7^ cells were seeded in 200 μl of PBS into the axillae of 7- to 8-week-old BALB/C nude mice to create the subcutaneous tumor model, and the tumor masses were observed and dissected after 30 days. The lung metastasis model was generated by injecting 2.0 × 10^6^ luciferase-labeled CNE2 cells in 100 μl PBS into mice through the tail vein. After 3 days, macrophage-depleting agents and controls were injected intraperitoneally every 3 days for 2 weeks, along with EVs for tail vein injection, until live imaging at five weeks. Liposomes of clodronate and phosphate buffer solution were purchased from ClodronateLiposomes.org (Haarlem, Netherlands). Clodronate was contained in the liposome formulation with a 5 mg/ml concentration. The success of the model was determined by measuring the photon flux throughout the mouse’s body each week using IVIS Lumina Series III. Weekly fluorescence measurements were performed on each mouse for 5 weeks to monitor lung metastasis. At the time of the lung metastasis measurements, the researchers were unaware of the means of intervention.

### Western blotting

Equal amounts of proteins were separated on 10% sodium dodecyl sulfate–polyacrylamide gel electrophoresis gel and transferred to polyvinylidene difluoride membranes. After blocking, the membrane was incubated with primary antibody (listed in Supplementary Data 2). Horseradish peroxidase (Santa Cruz Biotechnology, CA, USA)-conjugated secondary antibody was used and then visualized with ECL reagents.

### Immunohistochemistry (IHC)

The IHC was operated as previously stated [11]. The staining area was graded in four categories: 1 (zero to 25%), 2 (26 percent to 50%), 3 (51 percent to 75 percent), and 4 (>75 percent). Four grades were assigned to the staining intensity score: 1 (negative), 2 (weakly positive), 3 (moderately positive), and 4 (strongly positive). The product of the two staining scores was used to determine the final staining score, with scores 1–8 indicating low SCARB1 expression and scores 9–16 indicating high SCARB1 expression.

### Microscopy by transmission electron microscopy

First, we fixed the EVs with 2.5% glutaraldehyde. 5–10 μl of EVs solution was added dropwise to the copper mesh, adsorbed at room temperature for about 10 min, and excess liquid was carefully aspirated with filter paper. Then 10 μl of 2% phosphotungstic acid solution (pH = 6.5) was added dropwise to the copper net, the EVs were stained and processed for 2 min at room temperature, and the excess staining solution was carefully aspirated with filter paper, and the copper net was left to dry at room temperature. Finally, the observation was done on the machine with an observation voltage of 120 kV.

### The transwell assay

Migration assays were performed using Transwell inserts (Corning, 3422) with a pore size of 8 μm. In total, 5 × 10^4^ cells suspended in serum-free medium were added to the upper chambers, and a medium containing 10% fetal bovine serum was added to the lower chambers. After 16 h of incubation, the cells attached to the upper side were removed, and the cells attached to the underside of the membrane were fixed and stained with crystal violet. Digital images were obtained from the membranes, and five random fields were counted per chamber. Furthermore, we measured the number of cells by ImageJ. Lastly, the membranes were digitally imaged. ImageJ was also used to count the number of cells.

### Isolation and absorption of EVs

EVs were collected in the manner previously described [11]. Cell culture supernatants were differentially centrifuged at 500 × *g* for 10 min, 3000 × *g* for 60 min, and lastly 10,000 × *g* for 60 min at 4 °C to isolate EVs. A 0.22 μm filter was used before the supernatant was put into an ultracentrifuge tube. The supernatant was then centrifuged at 4 °C for 90 min at 100,000 × *g* (XPN-100, Beckman Coulter). NPC cells were cultured for 24 h in RPMI 1640 without FBS before collecting the culture supernatant. Phosphate-buffered saline was used to resuspend the isolated EVs. We labeled EVs with a PKH-67 labeling kit (Sigma-Aldrich, MINI67-1KT) and co-cultured them with macrophages for 2 h in order to detect EV absorption by recipient cells. Finally, macrophages nuclei were stained with DAPI. The photos were taken with a confocal microscope (Axio Observer, Zeiss, Göttingen, Germany).

### Assay for immunofluorescence

Firstly, freshly isolated mouse lung tissue is fixed in 4% paraformaldehyde for 6 h and transferred to 20% sucrose solution until the tissue sinks. Frozen sections were placed at room temperature for 30 min and washed 3 times with PBS for 5 min each. The sections were then closed with 2% BSA for 1 h at room temperature. The sections were then incubated overnight at 4 °C using a primary antibody (1:100). Then PBS was washed 3 times for 5 min each time. Next, incubating with fluorescent secondary antibody (1:200) for 1 h at room temperature and protected from light. Then, wash 3 times with PBS for 5 min each. Next, nuclear staining was performed using DAPI. PBS was washed 3 times for 5 min each time. Finally, the antifading agent was added, carefully sealing it with coverslips.

### Immunoprecipitation and mass spectrometry

According to the manufacturer’s instructions, cell extracts were prepared using the Co-Immunoprecipitation Kit (Thermo Scientific, 14321D). LC–MS assays were operated on an Easy nLC-connected Q Exactive mass spectrometer (Thermo Scientific). The MS data were examined with MaxQuant software. The database search results were filtered and exported at the peptide spectrum-matched and protein levels with a 1% FDR.

### ChIP assay

ChIP was performed following the manufacturer’s instructions using the Pierce Magnetic ChIP Kit (Thermo, 26157). To create DNA fragments ranging from 200 to 1000 bp, cells were sonicated and crosslinked with 1% formaldehyde. Antibodies against KLF9 (ABCAM, ab227920) or an isotype control IgG were then added to the precleared supernatant. Primers for HAAO/CYP1B1 promoter-binding sites were used in PCR. Their sequences were shown in Supplementary Data 2.

### Luciferase reporter assay

The luciferase reporter assay was performed by the previously described protocol [67]. To transfect the indicated plasmids and 1.5 ng pRL-TK Renilla plasmid, Lipofectamine 2000 Reagent (Thermo Fisher Scientific, Waltham, MA, USA, Cat. No. 11668019) was used (Ribo bio).

### Statistical analysis

GraphPad Prism Software was used to perform the calculations. Unless otherwise specified, results of at least three independent experiments are reported as means S.D. The Pearson correlation test was used to analyze the correlation in R. Survival was examined using Kaplan–Meier curves. One-way ANOVA and two-tailed Student’s *t*-tests were used to compare datasets with Gaussian distributions. Statistical significance was defined as *p* < 0.05.

### Supplementary information

Supplementary Data 1

Supplementary Data 2

Supplementary Data 3

Original Data File

## Data Availability

The data supporting our study’s findings are available from the corresponding author upon reasonable request.
