# Supplementary material for: SCARB1 in extracellular vesicles promotes NPC metastasis by co-regulating M1 and M2 macrophage function
Source: Cell Death Discov. 2023 Aug 29;9:323. doi: 10.1038/s41420-023-01621-9 (PMC10465564; doi:10.1038/s41420-023-01621-9)
Supplement: Supplementary file 1 — Supplementary Data 1 [file 41420_2023_1621_MOESM1_ESM.docx]

**Supplementary Data 1**

**Table S1. Clinical information of patients in the NPC study, related to Figure 3 and Table 1.**

| Age | Year of diagnosis | Gender (M: Male; F: Female) | TNM clinical stage | N | Survival status (Dead: 1; Alive: 0) | SCARB1 score |
| --- | --- | --- | --- | --- | --- | --- |
| 79 | 2005/2/9 | M | 3 | 1 | 1 | 12 |
| 78 | 2007/3/22 | M | 2 | 0 | 0 | 9 |
| 76 | 2005/12/9 | M | 3 | 1 | 1 | 8 |
| 70 | 2005/5/13 | M | 4 | 2 | 1 | 16 |
| 70 | 2006/11/12 | F | 3 | 1 | 1 | 12 |
| 70 | 2007/3/5 | M | 4 | 1 | 1 | 16 |
| 70 | 2007/1/10 | M | 3 | 1 | 1 | 9 |
| 70 | 2008/4/28 | M | 3 | 1 | 1 | 12 |
| 70 | 2008/5/19 | M | 2 | 0 | 1 | 2 |
| 70 | 2009/5/27 | M | 3 | 1 | 1 | 9 |
| 7 | 2009/11/3 | F | 2 | 0 | 0 | 2 |
| 69 | 2008/11/17 | M | 2 | 0 | 1 | 6 |
| 69 | 2009/6/25 | M | 2 | 0 | 0 | 6 |
| 68 | 2007/10/12 | M | 2 | 0 | 0 | 4 |
| 68 | 2008/10/30 | M | 4 | 2 | 1 | 16 |
| 68 | 2008/12/22 | M | 2 | 0 | 0 | 9 |
| 67 | 2007/3/20 | M | 2 | 0 | 0 | 8 |
| 67 | 2008/8/15 | F | 3 | 1 | 1 | 12 |
| 65 | 2006/2/3 | F | 3 | 0 | 1 | 6 |
| 65 | 2006/3/15 | M | 2 | 0 | 1 | 2 |
| 65 | 2006/4/19 | M | 2 | 0 | 1 | 6 |
| 65 | 2007/9/1 | M | 2 | 0 | 1 | 3 |
| 63 | 2009/5/22 | M | 2 | 0 | 1 | 4 |
| 63 | 2009/11/6 | F | 2 | 0 | 0 | 4 |
| 62 | 2007/10/19 | M | 4 | 1 | 1 | 12 |
| 61 | 2006/6/16 | M | 2 | 0 | 0 | 6 |
| 61 | 2008/1/31 | M | 3 | 1 | 1 | 16 |
| 60 | 2006/6/19 | M | 2 | 0 | 0 | 8 |
| 60 | 2007/1/16 | M | 4 | 1 | 1 | 16 |
| 60 | 2007/11/5 | M | 2 | 0 | 1 | 4 |
| 60 | 2007/5/21 | M | 4 | 2 | 0 | 9 |
| 60 | 2007/2/8 | M | 4 | 1 | 1 | 12 |
| 60 | 2008/4/3 | M | 2 | 0 | 1 | 4 |
| 60 | 2009/6/2 | M | 3 | 1 | 1 | 12 |
| 60 | 2009/6/18 | M | 1 | 0 | 0 | 3 |
| 59 | 2006/6/6 | M | 2 | 0 | 1 | 12 |
| 59 | 2007/7/6 | M | 2 | 0 | 1 | 4 |
| 59 | 2007/6/22 | M | 1 | 0 | 1 | 9 |
| 59 | 2009/2/20 | M | 4 | 2 | 1 | 16 |
| 59 | 2009/4/15 | M | 2 | 0 | 0 | 4 |
| 59 | 2009/9/6 | M | 2 | 0 | 0 | 12 |
| 58 | 2007/5/31 | F | 4 | 1 | 0 | 16 |
| 58 | 2007/7/29 | F | 2 | 0 | 1 | 4 |
| 58 | 2007/3/12 | F | 3 | 1 | 1 | 9 |
| 58 | 2008/9/22 | M | 1 | 0 | 0 | 4 |
| 58 | 2009/7/15 | M | 3 | 1 | 1 | 9 |
| 58 | 2009/10/9 | M | 1 | 0 | 0 | 4 |
| 57 | 2007/6/28 | M | 2 | 0 | 1 | 9 |
| 57 | 2009/11/3 | M | 3 | 1 | 0 | 9 |
| 57 | 2009/5/27 | M | 3 | 0 | 1 | 6 |
| 56 | 2005/6/21 | F | 1 | 0 | 0 | 1 |
| 56 | 2009/6/2 | M | 3 | 1 | 0 | 12 |
| 56 | 2009/2/8 | M | 2 | 0 | 0 | 4 |
| 55 | 2006/11/15 | M | 2 | 0 | 0 | 4 |
| 55 | 2007/8/3 | M | 4 | 1 | 1 | 16 |
| 55 | 2009/2/17 | M | 1 | 0 | 0 | 2 |
| 55 | 2009/6/17 | M | 2 | 0 | 1 | 3 |
| 55 | 2009/6/15 | M | 3 | 0 | 0 | 4 |
| 55 | 2009/7/28 | F | 4 | 2 | 1 | 8 |
| 54 | 2006/3/15 | M | 2 | 0 | 1 | 4 |
| 54 | 2007/11/22 | F | 3 | 1 | 1 | 9 |
| 54 | 2007/11/28 | M | 2 | 0 | 0 | 2 |
| 54 | 2008/5/30 | F | 3 | 1 | 0 | 9 |
| 54 | 2008/12/19 | M | 3 | 0 | 0 | 3 |
| 53 | 2005/11/29 | M | 3 | 1 | 0 | 12 |
| 53 | 2007/3/12 | M | 2 | 0 | 0 | 9 |
| 53 | 2009/12/1 | M | 2 | 0 | 0 | 6 |
| 53 | 2009/9/6 | M | 2 | 0 | 0 | 6 |
| 52 | 2006/10/24 | M | 2 | 0 | 0 | 4 |
| 51 | 2005/9/6 | M | 2 | 0 | 0 | 4 |
| 51 | 2006/9/8 | F | 2 | 0 | 0 | 6 |
| 51 | 2007/3/14 | F | 1 | 0 | 0 | 2 |
| 51 | 2007/4/6 | M | 1 | 0 | 0 | 1 |
| 51 | 2008/10/3 | M | 2 | 0 | 0 | 3 |
| 50 | 2005/7/26 | M | 1 | 0 | 0 | 1 |
| 50 | 2007/3/29 | F | 3 | 1 | 0 | 8 |
| 49 | 2005/12/16 | M | 2 | 0 | 0 | 2 |
| 49 | 2006/5/22 | F | 1 | 0 | 0 | 2 |
| 49 | 2007/10/16 | M | 3 | 1 | 1 | 12 |
| 48 | 2005/3/29 | M | 3 | 1 | 0 | 6 |
| 47 | 2006/10/25 | F | 2 | 0 | 0 | 6 |
| 47 | 2007/10/17 | M | 2 | 0 | 0 | 4 |
| 47 | 2009/7/24 | F | 2 | 0 | 0 | 6 |
| 46 | 2006/12/17 | M | 4 | 1 | 1 | 16 |
| 46 | 2007/5/3 | F | 2 | 0 | 0 | 2 |
| 46 | 2009/4/17 | M | 2 | 0 | 0 | 4 |
| 46 | 2009/8/21 | M | 3 | 1 | 1 | 9 |
| 45 | 2006/5/4 | F | 2 | 0 | 0 | 4 |
| 45 | 2008/6/8 | F | 3 | 1 | 0 | 12 |
| 45 | 2009/1/16 | M | 1 | 0 | 0 | 1 |
| 45 | 2009/4/9 | M | 4 | 1 | 1 | 12 |
| 44 | 2006/6/14 | M | 3 | 0 | 0 | 8 |
| 44 | 2007/7/3 | M | 2 | 0 | 0 | 2 |
| 44 | 2007/6/28 | M | 3 | 1 | 0 | 9 |
| 44 | 2007/2/8 | M | 4 | 2 | 1 | 12 |
| 44 | 2007/9/24 | F | 2 | 0 | 0 | 2 |
| 44 | 2007/10/24 | M | 2 | 0 | 0 | 4 |
| 44 | 2007/10/24 | M | 4 | 1 | 1 | 9 |
| 44 | 2007/11/21 | M | 4 | 3 | 0 | 16 |
| 44 | 2008/1/25 | M | 2 | 0 | 0 | 4 |
| 44 | 2009/6/19 | M | 2 | 0 | 0 | 3 |
| 44 | 2009/1/7 | M | 2 | 0 | 0 | 4 |
| 44 | 2009/7/9 | M | 3 | 1 | 0 | 12 |
| 43 | 2006/4/17 | M | 2 | 0 | 0 | 4 |
| 43 | 2007/3/21 | M | 4 | 1 | 0 | 16 |
| 43 | 2008/10/21 | M | 3 | 1 | 0 | 12 |
| 43 | 2009/5/31 | F | 2 | 0 | 1 | 4 |
| 42 | 2005/4/7 | M | 2 | 0 | 0 | 2 |
| 42 | 2006/10/16 | M | 3 | 1 | 1 | 8 |
| 42 | 2007/6/21 | M | 3 | 1 | 1 | 9 |
| 42 | 2007/12/9 | M | 2 | 0 | 0 | 3 |
| 41 | 2006/7/12 | F | 2 | 0 | 0 | 4 |
| 41 | 2007/10/16 | M | 3 | 1 | 0 | 12 |
| 41 | 2007/11/22 | M | 3 | 1 | 0 | 12 |
| 41 | 2009/2/13 | M | 2 | 0 | 0 | 4 |
| 41 | 2009/8/20 | F | 3 | 1 | 0 | 12 |
| 40 | 2006/12/21 | M | 1 | 0 | 0 | 3 |
| 39 | 2006/1/26 | M | 2 | 0 | 0 | 4 |
| 39 | 2007/1/2 | M | 3 | 0 | 0 | 3 |
| 39 | 2007/4/7 | F | 1 | 0 | 0 | 2 |
| 39 | 2007/7/16 | M | 3 | 1 | 0 | 9 |
| 39 | 2007/6/11 | F | 2 | 0 | 0 | 2 |
| 39 | 2008/5/13 | F | 2 | 0 | 0 | 3 |
| 39 | 2008/4/11 | F | 2 | 0 | 0 | 6 |
| 39 | 2008/4/12 | M | 1 | 0 | 0 | 2 |
| 39 | 2009/12/2 | F | 2 | 0 | 0 | 4 |
| 39 | 2009/10/7 | F | 2 | 0 | 0 | 4 |
| 38 | 2007/5/2 | M | 2 | 0 | 0 | 4 |
| 38 | 2008/5/28 | M | 2 | 0 | 0 | 6 |
| 38 | 2009/2/7 | F | 1 | 0 | 0 | 1 |
| 37 | 2005/10/10 | M | 1 | 0 | 0 | 2 |
| 37 | 2006/6/4 | M | 3 | 1 | 0 | 16 |
| 37 | 2008/6/3 | M | 3 | 1 | 0 | 12 |
| 37 | 2009/12/1 | M | 2 | 0 | 1 | 4 |
| 36 | 2006/12/15 | M | 3 | 1 | 0 | 9 |
| 36 | 2007/9/26 | M | 4 | 2 | 1 | 16 |
| 36 | 2009/3/17 | F | 2 | 0 | 0 | 6 |
| 35 | 2006/11/28 | M | 3 | 1 | 0 | 9 |
| 35 | 2007/7/29 | M | 4 | 2 | 1 | 12 |
| 35 | 2008/8/13 | M | 2 | 0 | 0 | 6 |
| 32 | 2007/2/11 | M | 2 | 0 | 0 | 2 |
| 32 | 2008/3/14 | F | 1 | 0 | 0 | 3 |
| 31 | 2009/8/5 | M | 2 | 0 | 1 | 6 |
| 25 | 2008/3/17 | M | 3 | 1 | 0 | 12 |
| 23 | 2008/3/12 | M | 2 | 0 | 0 | 4 |
| 23 | 2009/11/9 | F | 2 | 0 | 1 | 8 |
| 16 | 2005/8/3 | F | 2 | 0 | 0 | 4 |
| 12 | 2006/6/2 | M | 2 | 0 | 0 | 4 |
| 65 | 2008/5/19 | M | 3 | 1 | 1 | 12 |
| 43 | 2006/2/5 | M | 3 | 1 | 0 | 16 |

**Table S2. Clinical information of patients in the NPC study, related to Figure 1F and Figure 1G.**

| Age | Year of diagnosis | Gender (M: Male; F: Female) | TNM clinical stage | N | Survival status (Dead: 1; Alive: 0) | Pathological diagnosis |
| --- | --- | --- | --- | --- | --- | --- |
| 61 | 2009/4/1 | M | 2 | 0 | 1 | Squamous cell carcinoma |
| 78 | 2006/3/13 | F | 3 | 1 | 1 | Squamous cell carcinoma |
| 65 | 2005/10/10 | M | 3 | 1 | 1 | Squamous cell carcinoma |
| 25 | - | M | - | - | 0 | Mucositis |
| 38 | - | F | - | - | 0 | Mucositis |
| 42 | - | F | - | - | 0 | Mucositis |


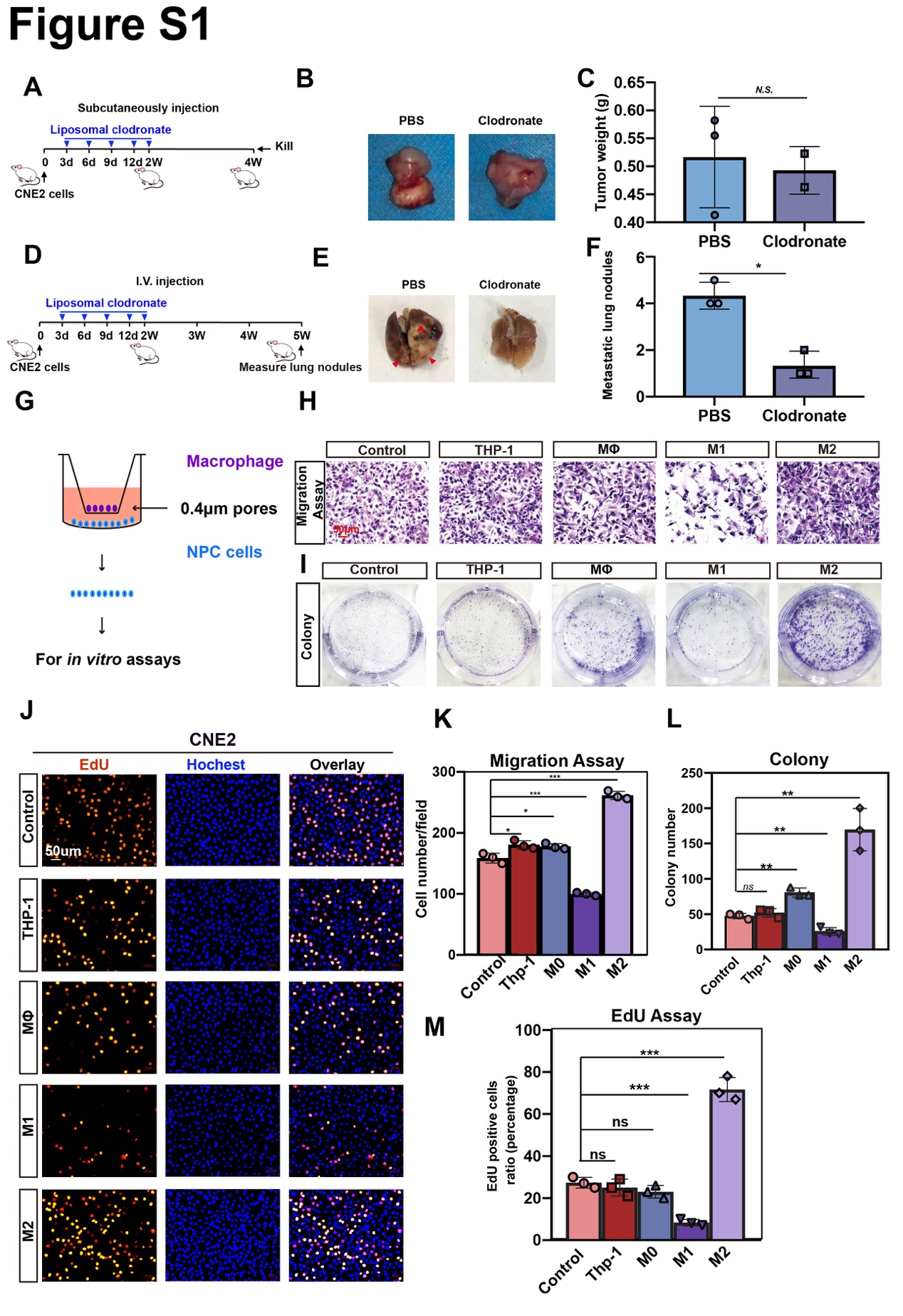


Fig. S1 The role of macrophages in the malignant biological behavior of NPC. A. Schematic diagram of subcutaneous tumorigenesis model and time point of intraperitoneal injection of macrophage depleting agent in nude mice. B. Diagram of subcutaneous tumors dissected out after 4 weeks of subcutaneous tumorigenesis in nude mice. C. Statistics of subcutaneous tumor weight in control and depleting agent groups. D. Schematic diagram of lung metastases injected into the tail vein of nude mice. E. Lung tissues with lung metastases. F. Statistics of the number of tumor nodules in lung tissues of control and depleted agent groups. G. Schematic diagram of co-culture of macrophages and tumor cells. H. Migration assay of CNE2 cells after co-culture with macrophages， the NPC cells in the control group were not co-cultured with macrophages. I. Colony formation assay. J. EDU assay. K-M. Migration assay, colony assay, and EDU Statistical plots of the experiments. All graphs show the mean ± SEM of at least three independent experiments. **P* < 0.05, ***P* < 0.01, ****P* < 0.001.


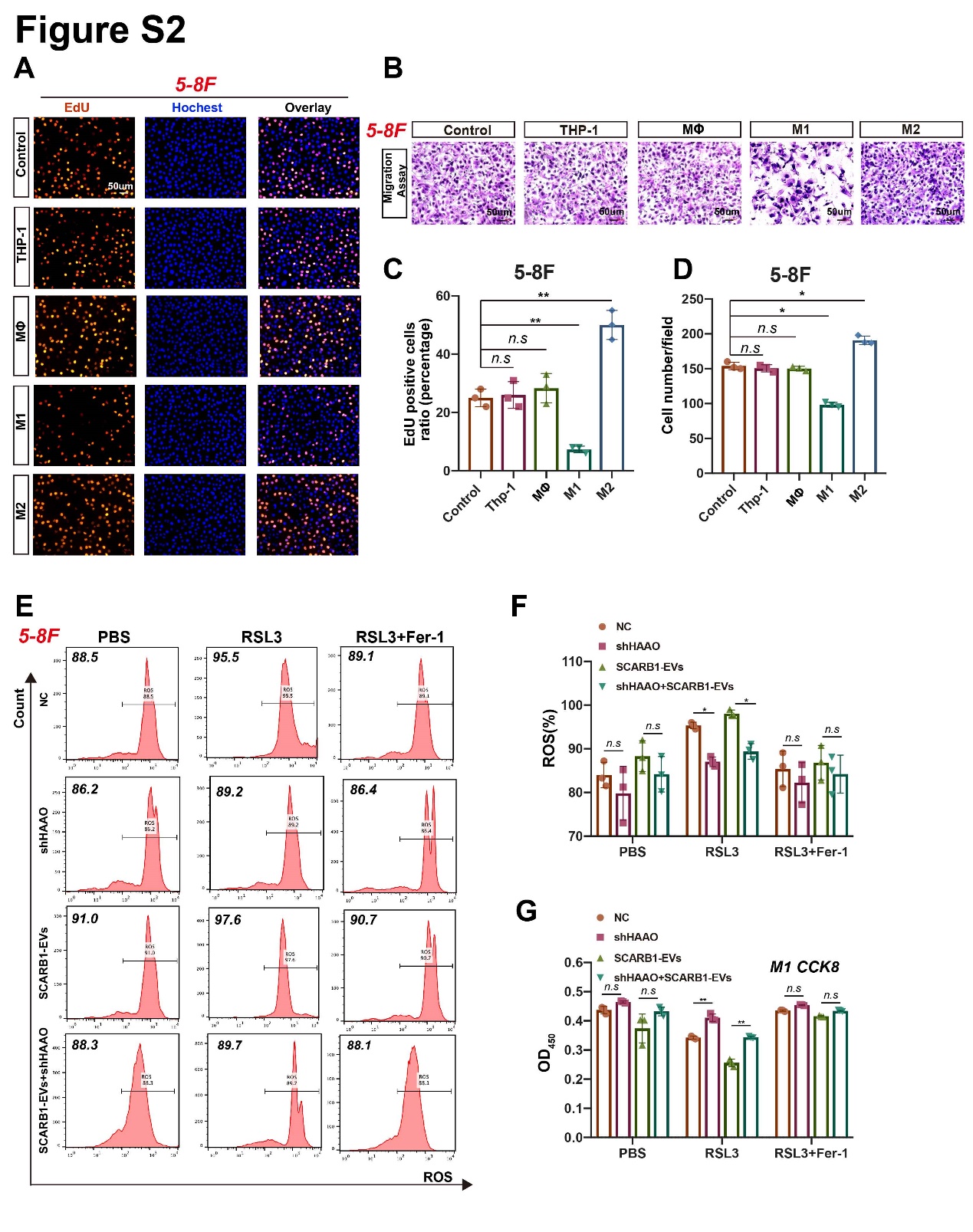


Fig. S2 A. EDU assay of 5-8F cells after co-culture with macrophages， the NPC cells in the control group were not co-cultured with macrophages. B. Migration assay. C, D. Statistical plots of the EDU assay and migration assay. E, F. Flow cytometry of ROS in macrophages (Two-way ANOVA). G. Cell death was estimated by CCK8 assay (Two-way ANOVA). EVs were derived from 5-8F cells. All graphs show the mean ± SEM of at least three independent experiments. **P* < 0.05, ***P* < 0.01, ****P* < 0.001.


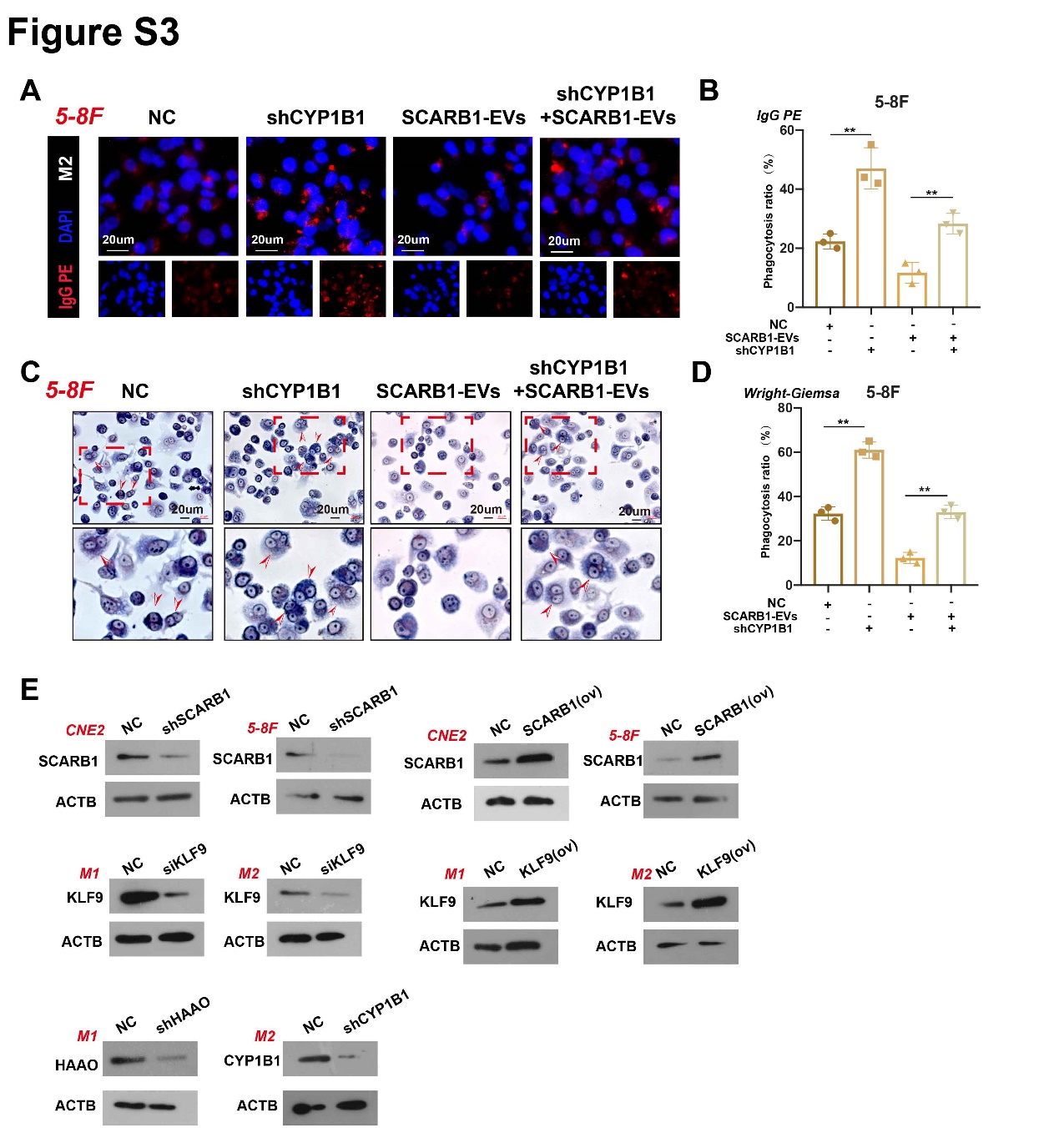


Fig. S3 A. Images of macrophages engulfing latex beads. EVs were derived from 5-8F cells. B. Statistics of devouring ability. C. Staining diagram of M2 macrophages with Wright-Giemsa. The arrow represents M2 macrophages after phagocytosis of 5-8F cells. D. Statistics of devouring ability (All graphs show the mean ± SEM of at least three independent experiments. ***P* < 0.01, ****P* < 0.001). E. Validation of the efficiency of the knockdown or overexpression vectors used in this study.
