# Supplementary material for: SCARB1 in extracellular vesicles promotes NPC metastasis by co-regulating M1 and M2 macrophage function
Source: Cell Death Discov. 2023 Aug 29;9:323. doi: 10.1038/s41420-023-01621-9 (PMC10465564; doi:10.1038/s41420-023-01621-9)
Supplement: Supplementary file 2 — Supplementary Data 2 [file 41420_2023_1621_MOESM2_ESM.docx]

**Supplementary Data 2**

The particular vector used to clone shRNA control and shRNA: hU6-MCS-Ubiquitin-EGFP-IRES-puromycin.

| SCARB1-RNAi (17930-1) | NM_005505 | cgGATTATTTGCTGAGCTCAA |
| --- | --- | --- |
| HAAO-RNAi (91690-1) | NM_012205 | ctGAGCAGTACAGAACAGGAA |
| CYP1B1-RNAi (16539-1) | NM_000104 | ccCAAGTCATTTAAAGTCAAT |

**Primers for HAAO/CYP1B1 promoter-binding sites were used in PCR.**

| **Serial number** | **Primer name** | **Sequence (5' to 3')** |
| --- | --- | --- |
| 1 | KLF9-HAAO-328-F1 | TTCTCAGGTGCTGGGACTTC |
| 2 | KLF9-HAAO-328-R1 | CCCTGGTGGACTTTGGTCT |
| 3 | KLF9-HAAO-220-F2 | CATTTTCCTCCTCCCCTACC |
| 4 | KLF9-HAAO-220-R2 | GGGAATCTCGGGGTGTTAAT |
| 5 | KLF9-HAAO-257-F3 | GGATATTTCAGACCCCCAAAA |
| 6 | KLF9-HAAO-257-R3 | TGACACATGAAGCACTGAGGA |
| 7 | KLF9-CYP1B1-259-F1 | GTTGTACCGAGCGTGGTTCT |
| 8 | KLF9-CYP1B1-259-R1 | TCCTCCGGGTTTTAAGGACT |
| 9 | KLF9-CYP1B1-286-F2 | GGAGCCGACTTTCCAGAAG |
| 10 | KLF9-CYP1B1-286-R2 | CTGAGATTTCCCGCGTAGAG |
| 11 | KLF9-CYP1B1-203-F3 | CCACCTCTTCCCTCGAGTTC |
| 12 | KLF9-CYP1B1-203-R3 | CGGCTCTGTGGTCTTCCTG |

Antibodies used for Western blot and IHC

| Antibodies | Manufacturer | Catalogue numbers | Dilution (WB) | Dilution (IHC) | Dilution (IF) |
| --- | --- | --- | --- | --- | --- |
| SCARB1 | ABCAM | ab217318 | 1/500 | 1/100 | 1/100 |
| HAAO | Proteintech | Cat No.12791-1-AP | 1/300 | 1/100 | 1/100 |
| CYP1B1 | ABclonal | A1377 | 1/600 | 1/100 | 1/100 |
| KLF9 | ABACM | ab227920 | 1/1000 | 1/100 | 1/100 |
| GPX4 | ABCAM | Ab125066 | 1/1000 |  |  |
| ACTB | Proteintech | 20536-1-AP | 1/1000 |  |  |
