# Supplementary material for: SCARB1 in extracellular vesicles promotes NPC metastasis by co-regulating M1 and M2 macrophage function
Source: Cell Death Discov. 2023 Aug 29;9:323. doi: 10.1038/s41420-023-01621-9 (PMC10465564; doi:10.1038/s41420-023-01621-9)
Supplement: Supplementary file 3 — Supplementary Data 3 [file 41420_2023_1621_MOESM3_ESM.docx]

**Supplementary Data 3**

**
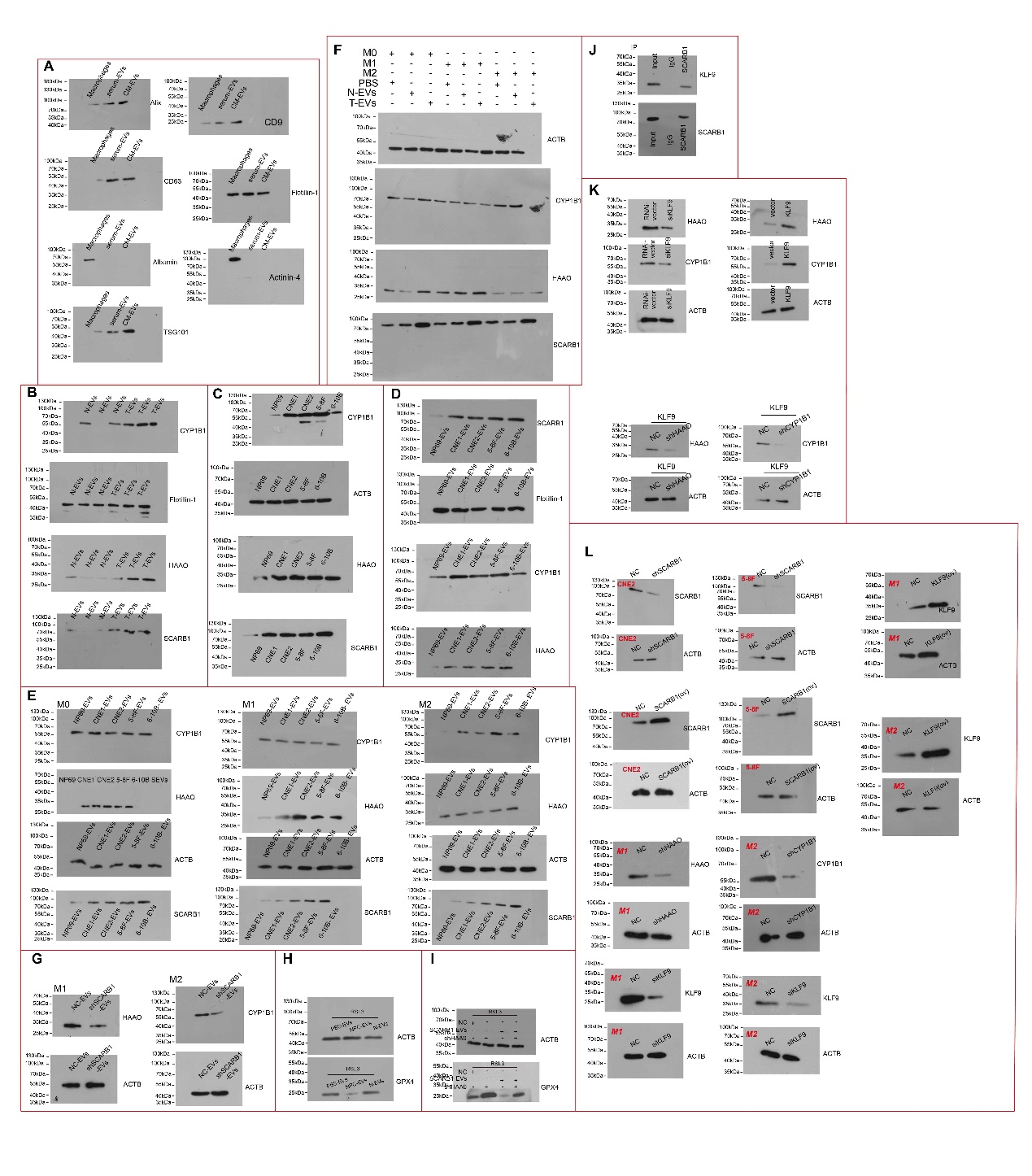
Supplementary Data 3** Uncropped blots for three independent experiments were presented in the figures. (**A**) Immunoblots of cropped blots as shown in **Fig. 1D**. (**B**) Immunoblots of cropped blots as shown in **Fig. 2B**. (**C**) Immunoblots of cropped blots as shown in **Fig. 2D**. (**D**) Immunoblots of cropped blots as shown in **Fig. 2E**. (**E**) Immunoblots of cropped blots as shown in **Fig. 2H**. (**F**) Immunoblots of cropped blots as shown in **Fig. 2J**. (**G**) Immunoblots of cropped blots as shown in **Fig. 2O**. **(H**) Immunoblots of cropped blots as shown in **Fig. 4G**.(**I**) Immunoblots of cropped blots as shown in **Fig. 4H**.( **J**) Immunoblots of cropped blots as shown in **Fig. 7B**. (**K**) Immunoblots of cropped blots as shown in **Fig. 7G**. （**L**）Immunoblots of cropped blots as shown in **Fig. S3E**.
