# Supplementary figures and images for: SCARB1 in extracellular vesicles promotes NPC metastasis by co-regulating M1 and M2 macrophage function
Source: Cell Death Discov. 2023 Aug 29;9:323. doi: 10.1038/s41420-023-01621-9 (PMC10465564; doi:10.1038/s41420-023-01621-9)

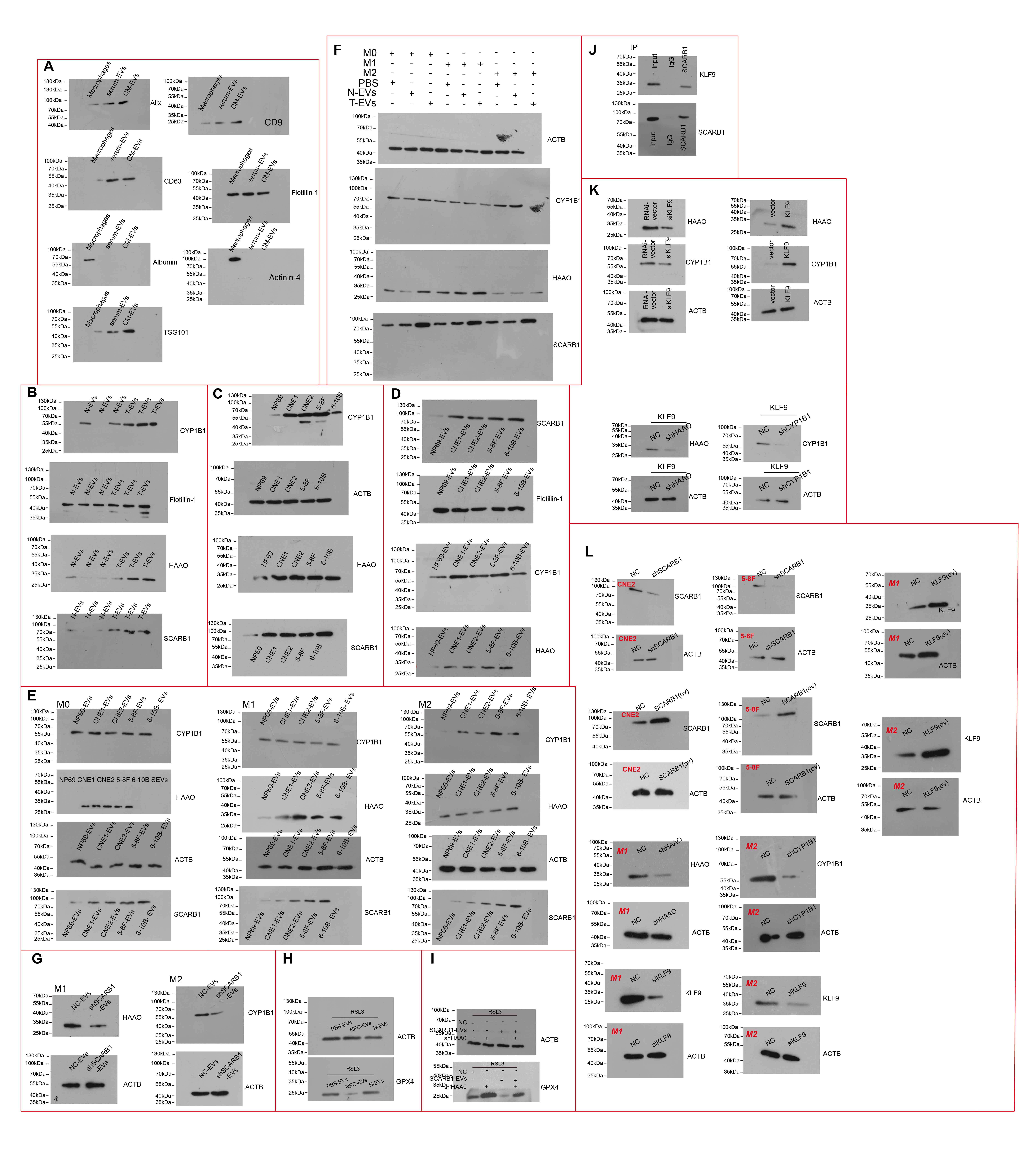

Supplement: Supplementary file 4 — Original Data File [file 41420_2023_1621_MOESM4_ESM.jpg]
